# Validity and Reliability of Inertial Measurement System for Linear Movement Velocity in Flywheel Squat Exercise

**DOI:** 10.3390/s23042193

**Published:** 2023-02-15

**Authors:** Sergio Maroto-Izquierdo, Kazunori Nosaka, Jesús Alarcón-Gómez, Fernando Martín-Rivera

**Affiliations:** 1 i+HeALTH, European University Miguel de Cervantes, 47012 Valladolid, Spain; 2 School of Medical and Health Sciences, Centre for Human Performance, Edith Cowan University, Joondalup, WA 6027, Australia; 3 Research Group in Prevention and Health in Exercise and Sport, University of Valencia, 46010 Valencia, Spain

**Keywords:** isoinertial, velocity-based training, encoder, validity, reliability

## Abstract

The aim of this study was to examine the validity and reliability of an Inertial Measurement System integrated into a secondary pulley (IMS) for determining linear velocity during flywheel squat exercises. Thirty-one male participants who were highly experienced in a flywheel resistance exercise training performed flywheel squat exercises with three incremental loads, and mean velocity (MV), mean propulsive velocity (MPV) and max velocity (Vmax) of the exercises were simultaneously recorded with a validated linear encoder and the IMS, in two different sessions. Validity was analyzed using ordinary least products regression (OLP), Lin’s concordance correlation coefficient (CCC), and Hedge’s g for the values from the linear encoder and the IMS. Test-retest reliability was determined by coefficient of variation (CV), Intraclass correlation coefficient (ICC), and standard error of measurement (SEM). Results showed a high degree of validity (OLP intercept = −0.09–0.00, OLP slope = 0.95–1.04, CCC = 0.96–0.99, Hedge’s g < 0.192, SEM = 0.04–0.08) and reliability (CV < 0.21%, ICC > 0.88, SEM < 0.08). These results confirm that the IMS provides valid and reliable measures of movement velocity during flywheel squat exercises.

## 1. Introduction

Velocity-based training (VBT) uses movement velocity to guide the prescription of training load during resistance exercise [1,2]. The use of this method of load prescription has been suggested to result in greater improvements in muscular strength and power through a more accurate intensity prescription in comparison to traditional methods based on the percentage of one-repetition maximum (1-RM) [3]. It has been proposed that VBT enables the objective determination of a given relative load [4], since exercise performance and neuromuscular fatigue can be precisely controlled during exercise, while providing information that can maximize training outcomes and reduce the risks of overtraining [5].

The foundation of VBT centers on the quantification of concentric linear velocity of each repetition (e.g., concentric mean velocity or mean propulsive velocity) with a linear position transducer [1], smartphone app [6], inertial movement sensor [7] or wearable devices [8]. In addition to the quantification of velocity, these devices can allow for the estimation of other kinetic and kinematic variables such as force and power [4]. Generally, the use of VBT focuses on the concentric velocity as training intensity is determined by the individual’s capacity to lift a weight (i.e., maximal concentric muscle strength) [9]. However, it is important to note that a higher absolute load can be used during the eccentric phase of a given resistance exercise [10]. As such, when attempting to optimize the loading strategy during resistance exercise, it is warranted to provide greater loads during the eccentric phase of any given exercises [11], either by prescribing high-speed stretch–shortening cycle exercises [12] or by providing an accentuated eccentric loading [13].

Flywheel technology represents one of the most-used exercise paradigms to provide eccentric-overload during resistance exercises [14]. Flywheel isoinertial devices use the flywheel principle [15]. Thus, to achieve an eccentric-overload, a maximum concentric muscle contraction is required to unwind the strap connected to the inertia wheel axis, spinning velocity into the flywheel system and accumulating kinetic energy, which occurs in response to increases in rotational speed. Once the concentric contraction is completed, the flywheel strap rewinds requiring a concentrated brief maximum eccentric contraction at the end of the eccentric phase to resist the forces that are applied in response to the rewinding of the strap [14,16]. It has been documented that isoinertial accentuated eccentric resistance exercise training is effective for muscle hypertrophy and increasing in muscle strength, muscle power, jumping performance and running speed in comparison with traditional resistance training [14,17]. It has been shown that flywheel resistance training is effective for enhancing sports performance [18], injury prevention [19] and rehabilitation and clinical context [20,21].

Due to the unique loading pattern associated with flywheel exercise, both concentric and eccentric muscle contractions can inform the training process by providing velocity and power data as well as the amount of eccentric overload [22]. Therefore, monitoring devices that provide kinematic data (i.e., angular speed) during flywheel exercises have been developed [23,24]. Rotary encoders are usually attached to the main axis of rotation, providing information about wheel angular velocity that can be used to estimate force and power when considering the moment of inertia [25]. However, the use of rotary encoders comes with some challenges as the inertia flywheel only changes its rotational direction between repetitions, which makes it difficult to differentiate between concentric and eccentric muscle actions and may smooth the oscillations of high-frequency velocity actions [7,26]. This may result in the linear encoder providing inaccurate kinetic and kinematic data [22,24].

To overcome these issues, recent studies have proposed the use of linear velocity to monitor concentric and eccentric velocity during flywheel exercises [7,22,26]. To this end, some studies have used an Inertial Measurement System (IMS) sensor (IonClinics & Deionic S.L, L’Alcudia, Spain) to control flywheel velocity [27,28]. The main difference between the IMS sensor and the rotatory encoder is its point of attachment, with the IMS being attached in a secondary pulley, which allows it to act like a linear encoder. Fundamentally due to its placement the IMS allows the differentiation of the kinematic data collected during the eccentric and concentric phases of the lift and a potential reduction of the possible kinematic dissonances between at the level of the axis of rotation and at the trainee’s level. The implementation of IMS sensors in flywheel devices could facilitate the correct prescription of velocity-based training as well as the acquisition of reliable data in force and power evaluation tests [22].

However, to the best of our knowledge, no study has analyzed the validity and reliability of this IMS sensor to establish the reproducibility of velocity measurements at different intensities and the accuracy in distinguishing the concentric and eccentric phases compared to a linear encoder. Therefore, the aim of this study was to evaluate the validity and reliability of the IMS to measure kinematic variables during squats performed on a flywheel device.

## 2. Materials and Methods

### 2.1. Study Design

Participants of the study participated in three sessions: one familiarization and two testing sessions separated by 72 h. During the testing sessions, participants were evaluated through an incremental loading test for three different inertial loads and performed 4 repetitions for each load. Data were collected simultaneity from two linear position sensors: (1) a linear-position transducer (LPT) which acted as criterion measure (1000 Hz sampling rate, T-Force, Ergotest, Murcia, Spain) and (2) the IMS (1000 Hz sampling rate, IonClinics & Deionic S.L, L’Alcudia, Spain). For the reliability analysis, the two fastest repetitions of each load set were used, so as not to artificially increase the reliability, they were determined separately for each device. For the validation analysis, the two fastest repetitions performed with each of the three loads were also used. The variables used for the reliability and validation analyses were mean velocity (MV), mean propulsive velocity (MPV) and max velocity (Vmax) that were simultaneously recorded with the LPT (i.e., criterion validity measure) and the IMS system.

### 2.2. Participants

Thirty-one healthy sports science undergraduate male students volunteered for the study (24 ± 2.9 years, height: 1.74 ± 0.92 m, body mass: 69.4 ± 15.4 kg, and body fat percentage: 16.5 ± 6.4%). They had, at least, one year of experience with flywheel training, and no history of neurological disorders or lower limb orthopaedical injuries. None of them were taking performance enhancing drugs, medications or other substances that could alter their performance during testing. Moreover, they were instructed to record and maintain their sleeping, eating, and drinking habits in the 48 h prior to each testing session. They were informed of the purposes and risks involved in the study before giving voluntary informed written consent to participate. The study procedures were performed in accordance with the principles of the Declaration of Helsinki and were approved by the local Institutional Review Board (H1421157445503).

### 2.3. Testing Procedures

During the familiarization session, participants practiced performing the flywheel squat exercise on the Epte Inertial Concept (Ionclionis and Deionic, LAlcúdia, Valencia-Spain) using three different inertial loads (2 sets of 4 repetitions with each load, with a 2-minute rest periods between sets), including the loads that would be used in testing sessions. During testing sessions, participants were evaluated through a randomized incremental loading test in which they performed 3 sets of 4 repetitions at three different intensities. The intensities used during testing were load 1: 0.02 kg·m^2^, load 2: 0.05 kg·m^2^, and load 3: 0.11 kg·m^2^ by different flywheels provided in the Epte Inertial Concept. The first repetition was always used to start the movement and to print speed into the flywheel system, and the other three repetitions were recorded for further analysis. A total of 837 repetitions were analyzed. During the next three repetitions, participants were requested to push with maximal effort (i.e., maximum possible concentric speed) through the entire concentric phase, which ranged from 90°-knee flexion to near full extension. To ensure that volunteers employed the same squat depth at each repetition, an adjustable tripod with a telemetric photocell (Microgate, Bolzano, Italy) was placed at the side of the flywheel device [27]. The telemetry photocell emitted a sound when participants reached 90° knee flexion. The randomized incremental loading test was replicated during testing session 2.

**Figure 1:**
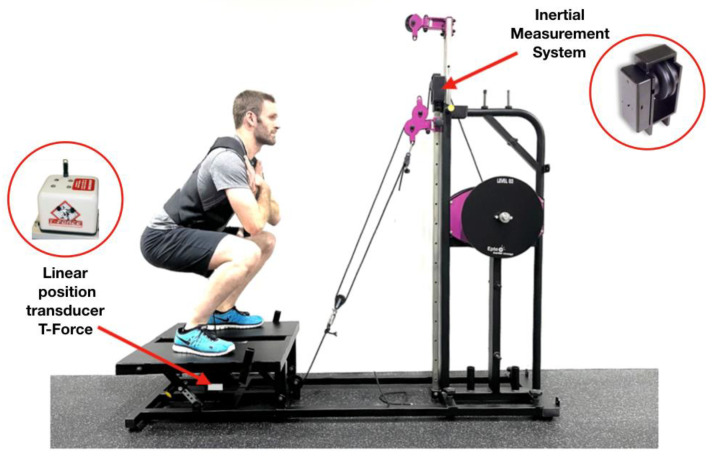
Squat exercise on the Epte Inertial Concept with a linear position transducer (LPT) and an Inertial Measurement System (IMS).

MV, MPV and Vmax for the concentric phase of the movement were collected simultaneously from the LPT (1000 Hz sampling rate, T-Force, Ergotest, Murcia-Spain) and the IMS sensor (1000 Hz sampling rate, IonClinics & Deionic S.L, L’Alcudia-Spain). The LPT was attached to the harness belt used for the exercise (Figure 1) and interfaced with a personal computer using a 14-bit resolution analog-to-digital data acquisition board and custom software. During each lift the instantaneous kinematic data were collected with a sample frequency of 1000 Hz. Reliability of the LPT used as criterion validity measure was previously reported [29].

The IMS sensor was a linear quadrature encoder (i.e., detecting the change of each phase when changing the input channel assigned for each phase of the movement) integrated into a secondary pulley through which the device cable passes, with the purpose to detect the linear movement of the cable and the direction of the movement, working as a traditional LPT (Figure 1). It collected data with a resolution of 48 points per revolution (4.8 mm) and a frequency of 1000 Hz. The two fastest repetitions (i.e., higher concentric MV) of each set, so as not to artificially increase the reliability, were determined separately for each device and used for further analysis. A total of 558 repetitions were analyzed for determining the reliability.

Before each session, a warm-up consisting of 5 min of cycling followed by 5 min of dynamic stretching exercises (i.e., forward leg swings, ankle dorsi- and plantar-flexion, side leg swings, high knees, heel flicks, squats and lunges) was performed, according to Cuenca-Fernández et al. [30]’s procedures (each exercise was performed for 20 s, and the entire set was repeated twice). Then, two sets of five continuous unloaded squats (i.e., non-jumping) interspersed by 30 s were performed at a rhythm of 2/2 (eccentric/concentric) tempo and 1/1 tempo, respectively [31].

### 2.4. Statistical Analyses

All study variables (MV, MPV and Vmax) were normally distributed according to the Kolmogorov–Smirnoff test. The concurrent validation of the IMS versus the criterion measure LPT (gold standard) was assessed using ordinary least products (OLP) regression [32]. Fixed bias (a significant systematic difference) was deemed present if the 95% confidence interval (CI) of the intercept did not include zero, while proportional bias (a significant proportional difference) was deemed present if the 95% CI of the slope did not include one [33]. Lin’s concordance correlation coefficient (CCC) was also used to assessing concurrent validity between the devices [34,35]. The criteria used to determine the strength of CCC were ‘almost perfect’ >0.99, ‘substantial’ between 0.95 and 0.99, ‘moderate’ between 0.90 and 0.95 or ‘poor’ <0.90 [36]. Hedges’ g effect sizes were also calculated to estimate the magnitude of differences between devices [37] and interpreted as trivial (<0.2), small (0.2–0.5), moderate (>0.5–0.8) or large (>0.8) [38]. Reliability was determined by the standard error of measurement (SEM), the coefficient of variation (CV) and the intraclass correlation coefficient (ICC). The criteria used to determine reliability were a SEM < 0.2, a CV < 10% and an ICC < 0.70 [39,40]. The validity and reliability analyses were performed for each load. A statistical significance was set at *p* < 0.05 level and a confidence limit was set at 95%. All statistical analyses, and accompanying plots, were performed using R software (The R Project for Statistical Computing, version 4.2.0).

## 3. Results

### 3.1. Validity

**Figure 2:**
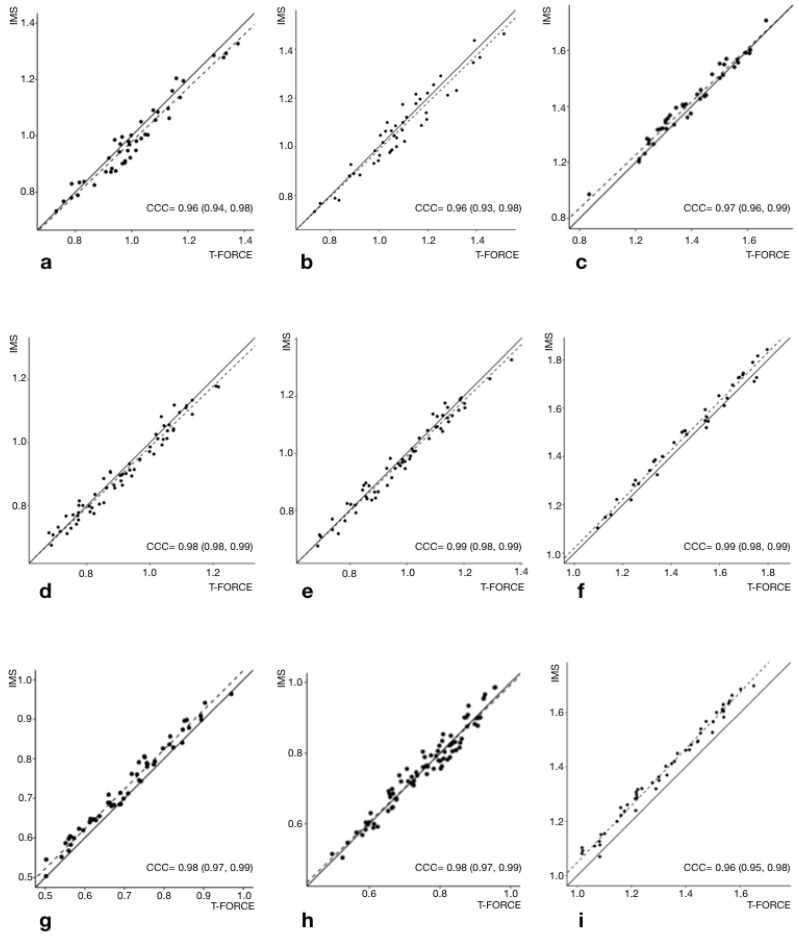
Lin’s concordance correlation coefficient (CCC) between the T-Force device (*X*-axis) and the IMS sensor (*Y*-axis) for mean velocity (MV: a, d, g), maximum velocity (Vmax: b, e, h) and mean propulsive velocity (MPV: c, f, i). MV—0.02 kg·m^2^ (**a**), MPV—0.02 kg·m^2^ (**b**), Vmax—0.02 kg·m^2^ (**c**), MV—0.05 kg·m^2^ (**d**), MPV—0.05 kg·m^2^ (**e**), Vmax—0.05 kg·m^2^ (**f**), MV—0.11 kg·m^2^ (**g**), MPV—0.11 kg·m^2^ (**h**), Vmax—0.11 kg·m^2^ (**i**). Data are shown in m/s, CCC concordance correlation coefficient = statistic value (Confidence Interval).

Descriptive statistics from both LPT and IMS sensor for each variable and load together with Hedges’ g effect size are included in Table 1. Hedges’ g effect size analysis showed no difference (<0.19) in any of the variables measured between both sensors. In addition, Table 2 includes the validity test results, showing a very high association between the measurements of both devices. The ordinary least products (OLP, intercept and slope data) regression showed that no fixed or proportional bias for any variable at any load. Similarly, Lin’s CCC showed values ranged from 0.96 to 0.99 for all variables studied, indicating that the degree of agreement between both measurement devices was almost perfect (Table 2, Figure 2).

### 3.2. Reliability

As shown in Table 3, all variables demonstrated good reliability between testing day 1 and 2, regardless of exercise intensity and the measurement device used (CV < 10% and ICC > 0.70). Both devices exhibited excellent relative reliability (ICCs > 0.9) and good absolute reliability (CV < 5%) for each variable studied regardless of the load tested (Table 3). In addition, the standard error of measurement (SEM) was lower than 0.09 for each load and for both LTP and IMS devices, indicating the high precision of the measurements.

## 4. Discussion

The results of this study showed that the IMS could be used as a valid and reliable system to control execution velocity during flywheel training. It was observed that the IMS sensor differentiated between concentric and eccentric phase; therefore, it is a valid and reliable tool for the measurements of MV, Vmax, MPV and ROM. The three loads tested showed Lin’s CCC values between 0.96 and 0.98 for MV, 0.96 and 0.99 for Vmax and 0.96 and 0.99 for MPV between the LPT criterion measures and the IMS (Table 2, Figure 2). Similarly, the IMS sensor showed significant ICC values (ranging from 0.88 to 0.95) for each load and output (Table 3). In addition, the standard error of measurement and CV were lower than 0.09 and 0.2%, respectively, for each tested load (see Table 3). Thus, this technology provides a better way to prescribe flywheel velocity-based exercise, hence providing information that can maximize training outcomes and reduce the risks of overtraining. In addition, the results of the present study permit the use of flywheel devices for testing when a IMS sensor is used.

Resistance training intensity has been traditionally prescribed relative to the maximum strength (based on the percentage of one-repetition maximum (1-RM)) [9], or based on an individual’s ability to lift the load at a given velocity [4]. However, in the case of flywheel technology, it is not possible to determine the 1-RM since there is not a maximum load that can be lifted [22]. Instead, during a flywheel exercise, the user needs to accelerate the inertia of the flywheel through a maximum concentric muscle contraction, and then apply a braking contraction to return the stored energy and invert the movement of the flywheel during the following eccentric phase of the exercise [22]. Therefore, considering the maximum nature of each concentric contraction together with the need to know the magnitude of the load during the eccentric contraction (i.e., eccentric:concentric ratio), concentric linear velocity is monitored during flywheel exercises [3,7,22]. Although concentric and eccentric velocity can be used to effectively prescribe exercise intensity [3], their accurate monitoring is quite complicated and requires the use of advanced technologies (e.g., 3D motion capture or quadrature encoders) that are not commonly available to practitioners.

This has prompted practitioners to use rotary encoders (integrated into the axis of the system), which provide information about the angular velocity of the flywheel [23,24]. However, a dissonance between what happens on the axis (i.e., angular velocity in a conical cylinder flywheel device) [41,42] and what happens at kinematic level while the participant is performing the exercise should be considered, which limits the use of velocity for such purposes. Although angular velocity can be converted to linear velocity mathematically, this relationship relies on a known radius (which can vary within some devices) and the lack of slack in cable/rope. As preliminary research has shown, rotary encoders function as a low-pass filter (i.e., smoothing the oscillations of high-frequency velocity actions [26], which is even accentuated when higher inertial loads are used, smoothing the instantaneous velocity record [43]. This could affect the calculations used to determine the mechanical power executed during the different phases of the movement and other estimated kinetic outcomes, altering not only the validity of these data, but also being responsible for the low reproducibility demonstrated when compared with LPT or force plates [23,24]. Moreover, rotary encoders do not allow the precise measurement of the concentric acceleration (or MPV in terms of velocity, impulse in kinetic terms) that the subject experiences during the accelerating phase of the concentric muscle contraction which determines the nature of the flywheel stimulus, and hence, the possibility of kinetically overloading the eccentric action [3]. It should also be considered that a rotary encoder does not physically differentiate between the concentric and eccentric phase of the movement [7]. This differentiation is usually performed by software prediction, which may lead to estimation errors (normally underestimating the concentric velocity). Moreover, in flywheel exercises performed with a load lower than 78% 1RM [5], rotary encoders will likely assign part of the concentric phase to the eccentric one, since participants usually stop applying force before completing the concentric contraction to avoid jumping, which may lead to erroneous estimates when controlling training load [2]. For all these reasons, it is necessary to validate a measurement instrument that can adequately differentiate between the concentric and eccentric phases during flywheel exercise, and thus can accurately quantify concentric linear velocity.

The results of the present study showed that the measurement of linear concentric velocity by means of an IMS sensor was a valid strategy for the control of flywheel exercise. Compared with a previously validated LPT device, which acted as a gold standard, Hedge’s effect sizes between 0.07–0.14 for MV, 0.13–0.14 for Vmax and 0.00–0.09 for MPV were observed at the three inertial loads tested. The validity of the IMS device has also been contrasted through the correlations of Lin’s CCC (ranging from 0.96 to 0.99 for all variables and loads) and OLP intercept (ranging from −0.09 to 0.02 for all variables and loads) and slope (ranging from 0.95 to 1.04 for all variables and loads) analysis, demonstrating an excellent correlation between the IMS and the LPT devices on MV, Vmax and MPV data (Table 2, Figure 2). Furthermore, it should be noted that these two statistical analyses (Lin’s CCC and OLP), despite having been used separately in previous validation studies [31,44,45], were for the first time applied simultaneously in the present study. Furthermore, this was the first study to graphically represent Lin’s CCC (Figure 2). Despite this, we cannot affirm that the IMS device has greater validity than other encoders, since the studies in which the validity of rotatory encoders [24,28] was studied have not applied a similar statistical treatment. Similarly, the IMS device showed excellent reproducibility between the two measurements analyzed for the measurement of MV (CV: 0.15–0.20%; ICC: 0.89–0.93; SEM: 0.04–0.05), Vmax (CV: 0.16–0.20%; ICC: 0.91–0.95; SEM: 0.06–0.09) and MPV (CV: 0.16–0.19%; ICC: 0.88–0.92; SEM: 0.04–0.06), showing a high similarity with the results obtained by the criterion measure LPT (Table 3). The present study showed similar values to those obtained by other articles about the validation and reliability of different devices for measuring exercise velocity in the variables studied (MV, MPV and Vmax), and similar results for SEM, CV, ICC and CCC, which coincide with the values considered as indicators of high similarity between results and high reliability [31,44,45].

However, some potential delimitations are worthy of mention. Despite the sample being homogeneous with respect to age, sex, strength level, and training experience, it is not known whether similar results would be found in women or individuals less exercise training experience. Furthermore, the inclusion of other parameters such as the measurement of the ground reactive force with a force plate system or the measurement of other kinematic parameters such as the eccentric velocity or the eccentric:concentric ratio is warranted. Future research is warranted to contribute to the knowledge and development of load-velocity profiles and their particularities in flywheel training by measuring linear velocity instead of angular velocity, as well as to develop measuring instruments that facilitate flywheel velocity-based training in order to individually monitor and adjust training volume and intensity in a better way to fit the needs of each participant.

## 5. Conclusions

In conclusion, the IMS was a valid and reliable tool for measuring concentric linear velocity during flywheel training. In comparison with a previous validated LPT, the IMS sensor provided almost the same values for MV, Vmax, MPV and ROM, as well as trivial Hedge’s g values for each outcome. In addition, the IMS presented good test–retest reliability for all loads tested between two different testing sessions. For the first time, this study provided evidence that the IMS accurately differentiated between concentric and eccentric phases of the movement during flywheel squat exercises. This in turn, enables practitioners to precisely estimate other kinetic values, such as eccentric:concentric ratio. In addition, it provides the feasible assessment of mean propulsive velocity, since this parameter is decisive in characterizing the nature of flywheel exercise. Therefore, the use of an IMS has the potential to be used as an avenue of intensity prescription, as well as provide valid and reliable data to monitor flywheel training and to control the training process.

## Figures and Tables

**Table 1:** Mean concentric velocity (MV), maximum velocity (Vmax) and mean propulsive velocity (MPV) at three different flywheel loads (0.02 kg·m^2^, 0.05 kg·m^2^, 0.11 kg·m^2^) measured by the T-Force and IMS, and Hedge’s g effect size between T-Force and IMS. All data are presented as mean (SD) of 31 participants.

Load	T-Force	IMS	Hedge’s g
**0.02 kg·m^2^**			
MV (m·s^−1^)	0.998 (0.152)	0.977 (0.148)	0.141
Vmax (m·s^−1^)	1.560 (0.274)	1.596 (0.265)	0.133
MPV (m·s^−1^)	1.072 (0.168)	1.050 (0.169)	0.091
**0.05 kg·m^2^**			
MV (m·s^−1^)	0.905 (0.140)	0.895 (0.134)	0.071
Vmax (m·s^−1^)	1.464 (0.208)	1.491 (0.210)	0.135
MPV (m·s^−1^)	0.971 (0.160)	0.962 (0.155)	0.061
**0.11 kg·m^2^**			
MV (m·s^−1^)	0.689 ( 0.119)	0.713 (0.119)	0.191
Vmax (m·s^−1^)	1.301 (0.191)	1.363 (0.201)	0.136
MPV (m·s^−1^)	0.764 (0.129)	0.764 (0.127)	0.003

**Table 2:** Validity test results by ordinary least products (OLP) regression (Intercept, Slope) and Lin’s concordance correlation coefficient (CCC) with 95% confidence interval (min, max) for mean concentric velocity (MV), maximum velocity (Vmax) and mean propulsive velocity (MPV) at three different flywheel loads (0.02 kg·m^2^, 0.05 kg·m^2^, 0.11 kg·m^2^) measured by the T-Force and IMS.

Variable	Intercept	Slope	Lin’s CCC
**0.02 kg·m^2^**			
MV	−0.00 (−0.06, 0.07)	1.02 (0.95, 1.08)	0.96 (0.94, 0.98)
Vmax	−0.09 (−0.18, 0.01)	1.04 (0.97, 1.1)	0.97 (0.96, 0.99)
MPV	0.02 (−0.05, 0.19)	0.99 (0.92, 1.06)	0.96 (0.93, 0.98)
**0.05 kg·m^2^**			
MV	−0.03 (−0.07, 0.02)	1.04 (0.99, 1.09)	0.98 (0.98, 0.99)
Vmax	−0.01 (−0.083, 0.042)	0.99 (0.954, 1.041)	0.99 (0.98, 0.99)
MPV	−0.02 (−0.06, 0.03)	1.03 (0.98, 1.07)	0.99 (0.98, 0.99)
**0.11 kg·m^2^**			
MV	−0.03 (−0.06, 0.001)	1.01 (0.97, 1.05)	0.98 (0.97, 0.99)
Vmax	0.00 (−0.04, 0.05)	0.95 (0.92, 1.00)	0.96 (0.95, 0.98)
MPV	−0.01 (−0.052, 0.018)	1.02 (0.98, 1,07)	0.98 (0.97, 0.99)

**Table 3:** Coefficient of variation (CV), Intraclass correlation (ICC) with 95% confidence interval (min, max), and standard error of measurement (SEM) values for three loads (0.02 kg·m^2^, 0.05 kg·m^2^, 0.11 kg·m^2^) for mean concentric velocity (MV), maximum velocity (Vmax) and mean propulsive velocity measured by the T-Force and IMS.

Variable	CV (%)	ICC (95%CI)	SEM
**T-Force**			
MV—0.02 kg·m^2^	0.150	0.90 (0.80,0.95)	0.048
MV—0.05 kg·m^2^	0.187	0.94 (0.89,0.97)	0.041
MV—0.11 kg·m^2^	0.206	0.92 (0.84,0.96)	0.038
Vmax—0.02 kg·m^2^	0.173	0.95 (0.90,0.98)	0.061
Vmax—0.05 kg·m^2^	0.201	0.94 (0.88,0.97)	0.071
Vmax—0.11 kg·m^2^	0.186	0.95 (0.89,0.97)	0.054
MPV—0.02 kg·m^2^	0.155	0.87 (0.76,0.94)	0.061
MPV—0.05 kg·m^2^	0.196	0.95 (0.89,0.97)	0.042
MPV—0.11 kg·m^2^	0.190	0.93 (0.85,0.96)	0.039
**IMS**			
MV—0.02 kg·m^2^	0.150	0.90 (0.80,0.95)	0.044
MV—0.05 kg·m^2^	0.183	0.89 (0.78,0.94)	0.054
MV—0.11 kg·m^2^	0.201	0.93 (0.85,0.96)	0.038
Vmax—0.02 kg·m^2^	0.163	0.95 (0.89,0.97)	0.059
Vmax—0.05 kg·m^2^	0.200	0.91 (0.82,0.95)	0.088
Vmax—0.11 kg·m^2^	0.187	0.95 (0.89,0.97)	0.057
MPV—0.02 kg·m^2^	0.156	0.88 (0.77,0.94)	0.059
MPV—0.05 kg·m^2^	0.194	0.92 (0.84,0.96)	0.053
MPV—0.11 kg·m^2^	0.187	0.90 (0.81,0.95)	0.045

## Data Availability

The data presented in this study are available on request from the corresponding author. The data are not publicly available due to participants’ privacy.
